# Educational Collaboration Between Russian-Born US Physicians and Russian Oncology Trainees in Evidence-Based Medicine: The Higher School of Oncology

**DOI:** 10.1200/GO.20.00546

**Published:** 2021-03-05

**Authors:** Ekaterina Baron, Michelle Sittig, Maxim Kotov, Ilya Fomintsev, Vadim Gushchin

**Affiliations:** ^1^ Department of Surgical Oncology, Mercy Medical Center, The Institute for Cancer Care at Mercy, Baltimore, MD; ^2^ Department of Head and Neck Cancer, N.N. Petrov National Medical Research Center of Oncology, St Petersburg, Russia; ^3^ The Cancer Prevention Foundation, St Petersburg, Russia

## Abstract

**PURPOSE:**

The 2-year Russian oncology residency focuses on diagnosis and treatment of malignancies but lacks evidence-based medicine (EBM) and patient communication skills (PCS) training. To overcome these educational disparities, the 5-year national program, the Higher School of Oncology (HSO), involving Russian expatriate physicians trained in the United States was established.

**METHODS:**

A retrospective study was conducted. Highly motivated oncology residents were enrolled in the program through the three-step selection process. US-trained Russian expatriate physicians acted as mentors. EBM skills were taught through weekly online journal clubs and clinical case presentations. PCS training included live seminars and simulations after journal clubs. EBM knowledge was assessed using Fresno test among newly enrolled and postgraduate year (PGY) 2-5 HSO residents. PCS were evaluated via simulation exam including two clinical scenarios (maximum score 100 each) among 17 PGY2 HSO residents and seven non-HSO trainees.

**RESULTS:**

Overall, 54 residents were enrolled over 5 years (8-13 annually); four were released from the program. The mean age was 24 ± 1 years, and 56% were females. Median scores of Fresno test were significantly higher among PGY 2-4 HSO residents compared with newly enrolled participants: 111 (IQR, 71-128) versus 68 (IQR, 42-84), *P* = .042; moreover, performance correlated with year of program participation (r_s_ = 0.5; *P* < .0001). PCS assessment score was significantly higher among HSO residents than non-HSO trainees: 71 (IQR, 58-84) versus 15 (IQR, 10-30) for scenario number 1 (*P* < .0001) and 78 (IQR, 71-85) versus 22 (IQR, 4-58) for scenario number 2 (*P* = .005), respectively.

**CONCLUSION:**

The involvement of Western-trained expatriates in remote education improves EBM and PCS among oncology trainees from their home country. This strategy can be useful in overcoming global medical education disparities in other specialties and in countries facing similar challenges.

## INTRODUCTION

Optimal cancer care is based on evidence-based medicine (EBM), a multidisciplinary medical approach implementing the best available evidence into patient care. Providing optimal cancer care in developing countries may be challenging not only because of limited material resources but also because of a lack of adequate oncology training.^1^ The absence of EBM and patient communication skills (PCS) training becomes a substantial obstacle for the realization of modern oncology essentials. Understanding and eliminating the educational deficiencies can significantly contribute to overcoming the problem of substandard oncological care.

**CONTEXT**

**Key Objective**
How can modern oncology competencies be successfully introduced into postgraduate training program in a developing country?
**Knowledge Generated**
Involvement of US-trained Russian expatriates in the training of young Russian oncologists is an effective strategy in teaching evidence-based medicine and patient communication skills. This approach seems to build long-term collaborations, providing a sustained educational program.
**Relevance**
Engagement of Western-trained expatriate physicians in the training of young oncologists from their home country can be a successful strategy for building a long-term educational program applicable to medical specialties.

Oncology training in Russia differs from Western postgraduate education and may be similar to other post-Soviet and developing countries. It includes only a 2-year residency program immediately following medical school, whereas many developed countries require 6-9 years of training, depending on the specialty.^2,3^ Limited access to international literature and a low frequency of English proficient physicians contribute to the Russian oncology community isolation from the world experience.^4^ The oncological curriculum based on Soviet-era materials primarily focuses on the diagnosis and initial treatment of tumors but lacks other important competencies including EBM and patient communication. Moreover, low popularity and shortage of EBM and patient-centered approaches (PCAs) among experienced Russian physicians decrease awareness of these necessary skills and create a hostile environment for young oncologists interested in exploring EBM.^5^ International collaboration is crucial and required for coping with these educational deficiencies.

Various initiatives have been recently launched by North American and European oncological societies to promote modern education and the EBM approach among young oncologists in developing countries. These initiatives include travel grants for European Society of Medical Oncology (ESMO) preceptorships, European School of Oncology (ESO) courses, and Global Oncology Young Investigator Award from ASCO, among others.^6-8^ However, the majority focus on short-term education of individuals rather than long-term large group learning. Therefore, the knowledge obtained by trainees remains on an individual level without institutional change. The routine involvement of international mentors can contribute to a solution; however, it may be limited because of cost and lack of motivation. On the contrary, the invitation of expatriates trained in the United States and Europe, who recognize the existing academic disparities in their home countries and have interest in improving the education of young physicians, can provide the opportunity for long-term systematic training.

A remote educational program, the Higher School of Oncology (HSO), was designed by Russian expatriate physicians practicing in the United States with the goal of introducing components of Western oncology medical education to Russian postgraduate oncological training. This study aims to evaluate the efficacy of the HSO program and suggests a model of remote training for young oncologists that can potentially be implemented in developing countries.

## METHODS

### Study Design

A retrospective study was conducted to analyze the efficacy of the remote educational program involving Russian expatriate physicians practicing in the United States in training young oncologists in Russia.

### Participants and Settings

**FIG 1:**
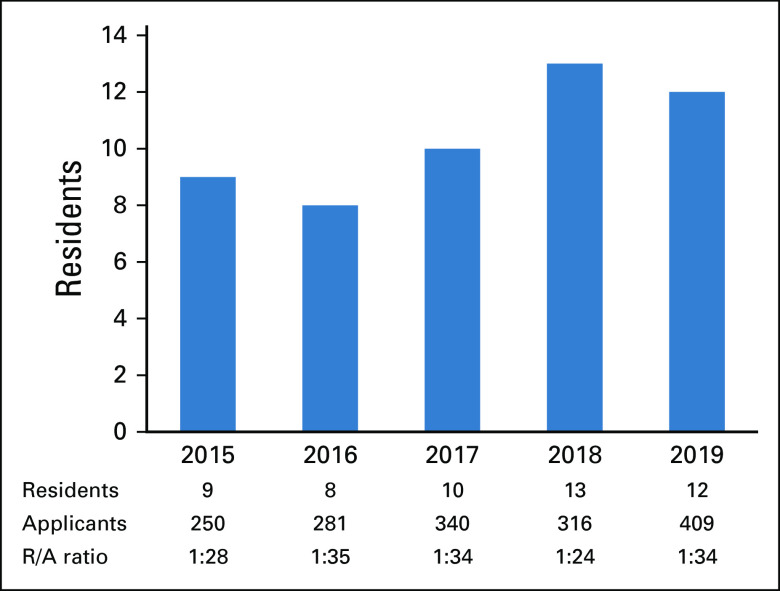
Annual application and enrollment to HSO program. HSO, Higher School of Oncology.

Medical school and general surgery internship graduates were enrolled in HSO through a highly competitive three-step application process including assessment of medical school performance, curriculum vitae review, medical knowledge test, essay writing, and personal interview. The annual enrollment was 8-13 residents from 250 to 830 applicants across Russia, Ukraine, Belarus, and Kazakhstan (Fig 1). Enrollees entered the 2-year oncology residency program located in Moscow or Saint-Petersburg hospitals.

### Data Source and Variables

Participant data were collected prospectively between April 2015 and January 2020. English proficiency was estimated according to the Common European Framework of Reference for languages where A1/A2 is a basic user, B1/B2 independent user, and C1/C2 proficient user.^9^ Medical school performance was evaluated by grade point average with a maximum of 5.0.

### Intervention

#### The HSO Program.

**FIG 2:**
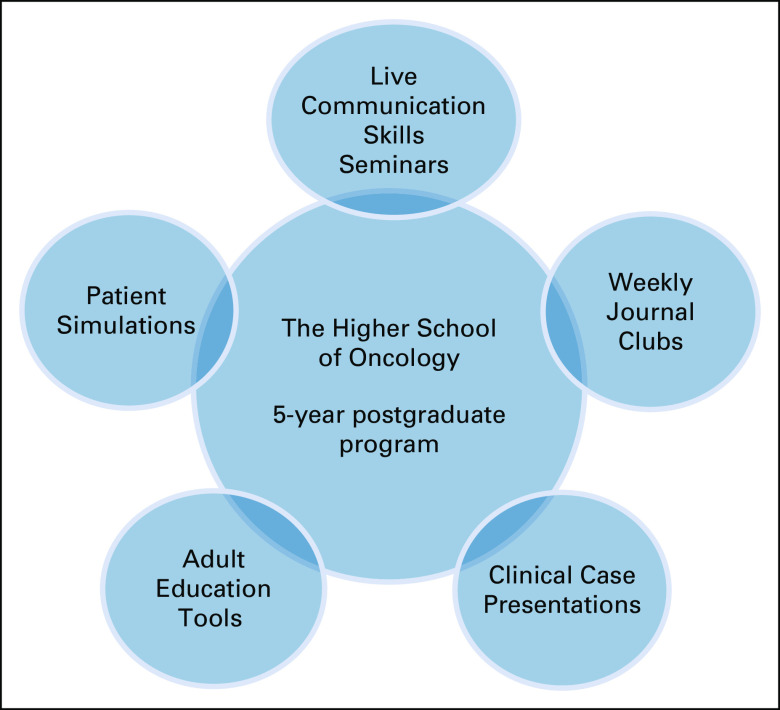
The HSO essential components. HSO, Higher School of Oncology.

The 5-year national program “The Higher School of Oncology” was initiated in 2015 by a US practicing surgical oncologist, trained in Russia and then in the United States. The program partnered with a Russian nonprofit cancer awareness organization, dedicated to promoting EBM that provided local technical and organizational support including curriculum, networking, and student financial resources. The curriculum consisted of topics ranging from basic tumor biology to specific concepts in surgical, medical, and radiation oncology. Russian-speaking physicians from various US clinics specializing in medical, surgical, and radiation oncology, pathology, and palliative care instructed students on critical reading of scientific literature, clinical decision making, conducting scientific work, and patient communication through online videoconferences with journal clubs and resident case presentations. Training was conducted in both English and Russian languages with oral discussions in Russian, and all appraised articles and educational sources in English (Fig 2).

#### Online Journal Club.

Two-hour weekly journal clubs were held each weekend that included articles focused on key oncological trials published within the past 15 years. After article appraisal, participants discussed various ways to implement scientific findings from trials to the local healthcare system. Examples included introduction of sentinel lymph node biopsy for melanoma and peritoneal washings for gastric cancer. Through simulated scenarios, participants explored the necessary steps in the organizational process, engagement of multidisciplinary team members, and advantages of the method for both patients and the cancer care team.

#### Communication Skills Training.

Patient-centered skills were taught through live seminars conducted among postgraduate year (PGY)-1 program residents during mentor visits to Russia and via mentor supervised simulations among residents after online journal clubs. Communication skills included training on empathy, delivery of bad news regarding poor prognosis and iatrogenic injuries, explaining complicated figures, research results, and other complex issues using simple nonmedical language. The seminars had theoretical and simulation components. Theoretical basis for the seminars originated from the preparatory materials for the United States Medical License Exam (USMLE) Step II Clinical Skills exam and seminal papers on the topic. Simulations of common scenarios included explanation of risks and benefits of adjuvant chemotherapy, conducting preoperative conversations, and delivery of bad news to patients, which were taught according to SPIKES protocol.^10^

#### Transition from Student to Teacher.

In addition to the above classes, PGY 3-5 HSO residents conducted their own journal clubs and several short courses for junior residents on a variety of subjects including biostatistics, academic writing, and system-based practice (SBP) in oncology and medical public relations in social networks. HSO residents also taught similar courses to medical students, residents from other programs, and attending physicians with the aim of promoting EBM and a more PCA among Russian physicians.

#### Efficacy Assessment.

EBM knowledge after program introduction was assessed using the Fresno test, which scores 12 open-ended questions with standardized grading rubrics, with a maximum total score of 212.^11^ A blinded assessment of recently enrolled residents (before program exposure) and PGY2-5 HSO residents was performed by two independent EBM experts. Communication skills were evaluated with actor-simulated exams and participants blinding by an independent private education group engaged in teaching communication skills. Each resident obtained two of four available clinical scenarios: three cancer-related scenarios (breast, colon, and gastric) to test oncology-specific communication approaches and one cardiac scenario to assess basic communication skills. Scenarios were rated among 17 PGY-2 HSO residents (over 2 years) and seven non-HSO young oncologists with a maximum score of 100, for each scenario. Criteria analyzed included the ability to reduce patient anxiety; ask open-ended questions; identify patient concerns; actively listen and provide empathy; assess patient values and preferences; provide relevant patient information; maintain an appropriate pace of information delivery, presenting options with pros and cons; and make shared decisions.

### Statistical Analysis

Statistical analysis was conducted using SPSS v.23.0 software. Participants and intervention characteristics were analyzed. Continuous variables are presented as means or medians and categorical variables as proportions. Differences between groups in the Fresno test and communication skills exam scores were assessed using the unpaired Mann-Whitney test. The Kruskal-Wallis one-way analysis of variance for pairwise comparisons of medians was used to assess differences in the Fresno test performance among PGY1-5 residents. Correlation between the Fresno test score and participation year in the HSO was analyzed with the bivariate Spearman rank correlation test. The results are presented in boxplots. The *P*-value < .05 was considered statistically significant.

## RESULTS

Over 5 years, 54 residents were selected for HSO participation. Four residents were released from the program during their first year because of low attendance (n = 2), low academic performance (n = 1), and failure to comply with organizational standards (n = 1).

### Participant Characteristics at Enrollment

Participant characteristics are presented in Table 1. The mean age at enrollment was 24 ± 1 years. There were 24 (44%) males and 30 (56%) females. Among 54 enrolled residents, 11 (20%) were internship graduates. Distribution of resident specialties included 33% (n = 18) surgical oncology, 46% (n = 25) medical oncology, 6% (n = 3) radiology oncology, 6% (n = 3) pediatric oncology, and 9% (n = 5) oncology pathology.

**TABLE 1:**
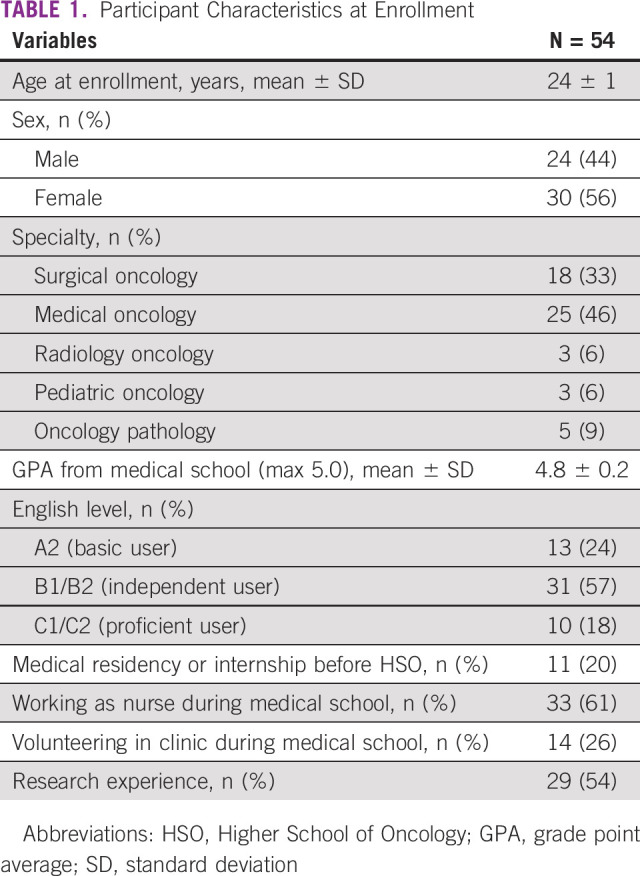
Participant Characteristics at Enrollment

The mean medical school grade point average was 4.8 ± 0.2. Enrollment English proficiency levels were 13 (24%) A2, 31 (57%) B1/B2, and 10 (18%) C1/C2. More than half (54%) of the participants had previous research experience. Thirty-three (61%) residents worked as nurses, and 14 (26%) volunteered in clinics during medical school.

### Journal Club and EBM Knowledge

Overall, 195 journal clubs were conducted over 5 years, averaging 39 sessions annually. The majority of journal clubs were dedicated to surgical and medical oncology trials (87%). Other topics included radiology (6%), palliative therapy (3%), oncological pathology (3%), and hematology (1%).

**FIG 3:**
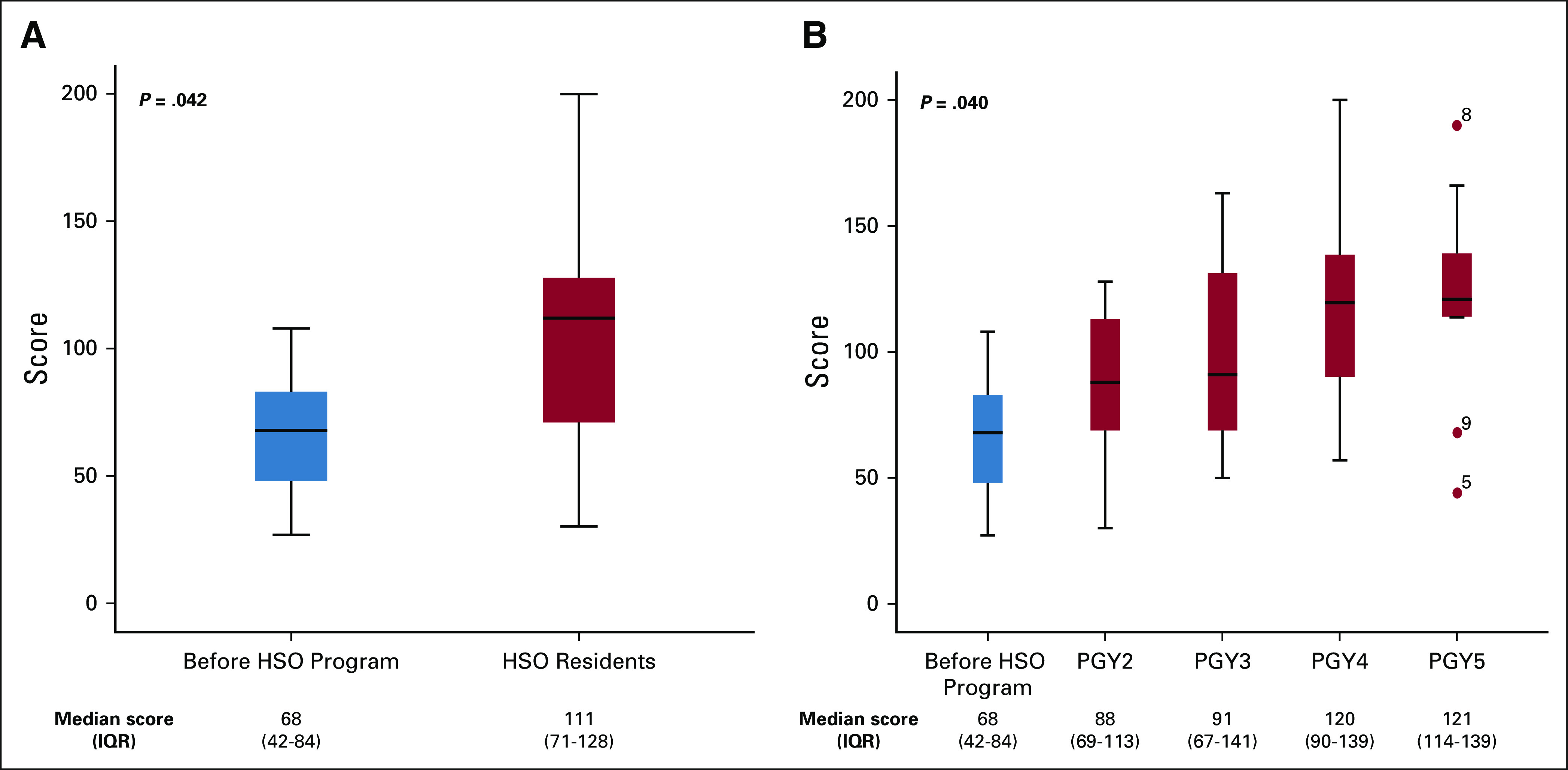
Boxplots of EBM knowledge assessment results before and during HSO program: (A) total and (B) by PGY. EBM, evidence-based medicine; HSO, Higher School of Oncology; PGY, postgraduate year.

EBM knowledge significantly improved with HSO program advancement. Fresno test scores increased from 68 (IQR, 42-84) before program exposure to 111 (IQR, 71-128) in PGY2-5 HSO residents, *P* = .042. There was EBM knowledge improvement with length of participation: newly enrolled PGY1 (before HSO) median score 68 (IQR, 42-84), PGY2 88 (IQR, 69-113), PGY3 91 (IQR, 67-141), PGY4 120 (IQR, 90-139), and PGY5 121 (IQR, 114-139), *P* = .040. Fresno test performance correlated with year of program participation (PGY1-5) (r_s_ = 0.5; *P* < .0001) (Fig 3).

### Communication Skills

**FIG 4:**
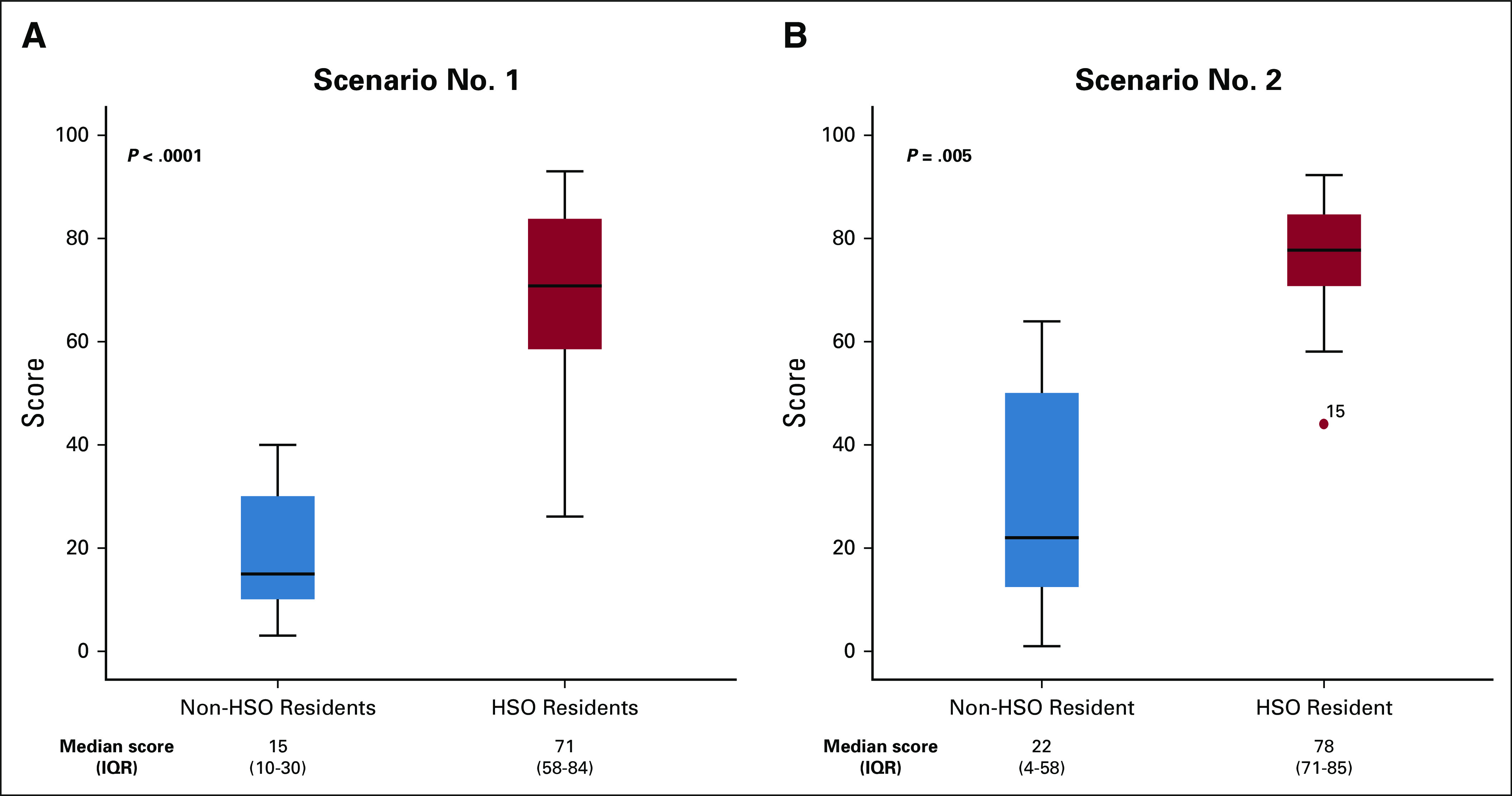
Boxplots of communication skills test results. HSO, Higher School of Oncology.

The median score for scenario number 1 was significantly higher in HSO residents than in non-HSO residents: 71 (IQR, 58-84) and 15 (IQR, 10-30), respectively, *P* < .0001. The performance in scenario number 2 was also better in HSO residents compared with that in non-HSO residents: median 78 (IQR, 71-85) versus 22 (IQR, 4-58), *P* = .005 (Fig 4).

## DISCUSSION

This study presents the results of the remote educational program “Higher School of Oncology,” which supplemented the standard oncology residency curriculum with the aim of promoting EBM and a PCA among young Russian oncologists. The program enrolled highly motivated and competitive residents and included weekly 2-hour journal clubs, PCS seminars, regular patient simulations, and short courses dedicated to academic writing, biostatistics, and leadership. After 5 years of HSO existence, motivation to participate did not decline in mentors, residents, or local organizers. EBM knowledge of residents significantly improved after program exposure and directly correlated with the length of participation. Communication skills after 2 years of HSO participation were significantly better than those in non-HSO young oncologists. These findings suggest that the remote educational program conducted by US-trained expatriates is sustainable and efficient in EBM training and PCS among young physicians from their home country.

The necessity of introducing an HSO program to Russian postgraduate education was developed from the deficiencies of Russian oncology education. Modern oncology training should include epidemiology, critical appraisal of medical literature, research literacy, implementation of best available evidence into clinical decision making, and PCS along with standard and comprehensive theoretical basis.^1,12^ Although many of these proficiencies are mandatory for postgraduate oncology training in well-resourced countries, they are lacking in the Russian curriculum. In addition, the short duration of oncology medical residency immediately after medical school, without practice in general specialties (eg, internal medicine and general surgery), poor research culture, and low prevalence of English-reading mentor physicians impede obtaining these skills by oncology residents.^5,13-15^ The absence of an appropriate learning environment might also be an obstacle for acquiring EBM and PCA concepts. Despite rising interest in evidence-based practice among the progressive segment of the Russian medical community, this approach is still unpopular among practicing physicians, who make up the majority of mentors for young doctors.^16^ International grants and studying abroad can raise awareness for young physicians about the importance of EBM and PCA and facilitate obtaining these skills; however, their implementation in real practice might be challenging without a mentor using these concepts, the support of peers, and a welcoming learning environment. We attempted to overcome existing barriers with role models of successful US-trained compatriot mentors, team building, and group practice of skills that can be taught remotely, such as EBM and efficient patient communication. International cooperation with practicing physicians combined with developing a core of young professionals with modern knowledge and a fresh perspective may create an appropriate environment and lead to a positive shift of the educational paradigm.

Journal club and case presentations were selected as main tools for teaching EBM skills and key oncology concepts. Studies show that regular participation in a journal club during postgraduate education improves critical thinking and epidemiological skills.^17-19^ It is challenging to introduce a journal club in circumstances where the majority lacks English reading skills, and there is a shortage of mentors experienced in EBM. In our program, weekly online journal clubs were conducted by international experts. The Fresno test assessment showed significant improvement of EBM skills even after the first year of training. The median score of HSO residents was 111 (IQR, 71-128), which is lower than expert level (mean, 147.5) in the study by Ramos et al^11^ of Fresno test validation. This can be explained by the difference of baseline levels in participants. The mean score of novice students in their study was 95.6, whereas the median score of our residents before HSO was only 68 (IQR, 42-84). Despite enrollment of highly selective participants at the beginning of the program, the majority were unable to explain basic concepts and principles of EBM including the primary end point, CI, or intention-to-treat analysis, indicating an obvious deficit of clinical epidemiology knowledge. However, by the end of the first year, residents were able to critically appraise articles and, by the third year, started mentoring junior residents by conducting journal clubs. This may explain the continuing Fresno test performance improvement with every year of program participation, since teaching others during independent conduction of journal clubs may allow honing of EBM skills. These findings suggest that online journal clubs moderated by international experts can be effective in areas with a lack of local EBM experts.

Patient communication is another core professional competency, which is not mandatory in Russian medical school and postgraduate curriculum.^20^ Attaining communication skills by young physicians usually occurs in a passive watch and copy way during observation of more experienced colleagues. Along with a limited efficacy and the absence of feedback, this approach leads to absorption of a paternalistic model, which remains widespread in Russian clinics.^21^ To address these issues and promote PCA, patient communication training was included in the HSO program. Live seminars were conducted annually during US mentors’ personal visits to Russia, followed by regular simulations during and after online journal clubs to demonstrate and practice necessary techniques. Subsequent assessment with two clinical scenarios showed improved communication skills among PGY2 HSO residents compared with non-HSO young physicians. After 3 years, two HSO residents created an online patient communication course for Russian-speaking physicians and a Telegram channel, “How to say,” with the same focus with more than 3,400 followers.^22,23^ Based on these findings, we can conclude that teaching patient-centered communication to young physicians not only improves their own skills but also promotes the spread of PCA within an overall paternalistic medical community.

Implementation of EBM requires skills of SBP. Otherwise, it may be challenging to introduce new practice based on evidence in the healthcare system that is structured differently from the one from which the evidence was obtained. SBP refers to the knowledge that each individual practice is a part of a larger healthcare system and the physicians’ ability to use the system to maintain and develop patient care.^24^ SBP was defined as one of the six core competencies of medical residency graduates defined by the Accreditation Council for Graduate Medical Education (ACGME) and the American Board of Medical Specialties (ABMS) in 1998.^25,26^ However, SBP skills remain abstract and there is no clear consensus on how they should be taught and evaluated. HSO taught these skills through discussing essential steps when introducing scientific evidence and protocols into clinical practice following journal clubs. These skills are crucial for young Russian oncologists as, traditionally, the initiative to implement new strategies in Russian clinics comes from administration, whereas the majority of physicians believe that they are not entitled to initiate change. In such conditions, the absence of SBP skills may lead to the disutility of obtained knowledge by HSO residents. Teaching leadership skills and SBP is necessary along with EBM and PCA in circumstances where these approaches are not widespread.

A sustainable remote program required several key components. First is the involvement of Russian expatriate physicians with US oncology training, who were therefore aware of their home country's education culture and had substantial modern knowledge to apply to medical education discrepancies. Furthermore, the mentors' dedication to advance oncology training in their homeland provided the motivation necessary to maintain a complex long-term program. Second, a rigorous three-step selection application process was implemented to identify highly motivated participants and those who would best benefit from this intense program. We believe that such candidates have the ambition and career potential to promote the obtained skills within the medical community. Four participants (7%) were terminated during PGY1, which encouraged us to conduct self-assessments to improve the selection process and education. Third, a close partnership with a nonprofit cancer awareness organization localized in St Petersburg, Russia, and particularly, its leader provided an effective on-site administration to support these processes. We believe that collaboration of leaders applying their expertise in the areas of medical education and organizational strategies along with enrollment of highly motivated students is crucial for building a solid and efficient remote educational program.

Despite overall positive outcomes, there were a number of challenges during program development. Early in the program, technical difficulties related to internet speed and connection obstructed videoconferencing. To prevent these issues, a convenient platform for video communication and high-quality equipment, such as cameras and microphones, were needed. Although convenient, it took time for participants to adjust and become familiar with this new virtual communication format. Additionally, the 7-8 hour time zone difference between Russia and the United States resulted in limited scheduling options for communication and videoconferencing. Because of scheduling constraints, seminars were often only possible during the weekends, requiring high dedication of both mentors and trainees.

The status quo bias of the local medical community proved to be the biggest challenge for all HSO participants. Teaching EBM skills and efficient patient communication is challenging in circumstances where medical professionals are unaware that these skills are beneficial and lacking. This can degrade mentor and participant motivation and provoke a negative response in the local community toward the new educational strategy. After the first 6 months, HSO residents faced a confrontational reaction from part of the local medical community in response to attempts of implementing EBM and shared decision making. This reluctance to implement new strategies might be an expected behavior previously described after EBM introduction.^27-29^ In addition to resistance, the absence of local role models practicing EBM and PCA was discouraging for HSO residents and mentors. However, the involvement of regional like-minded physicians, EBM popularization in social networks, and training residents to teach these skills have helped to better convey the main project idea and mitigate initial antagonism. After 2 years of program initiation and implementation, rejection changed to keen interest within the medical and patient community, which led to invitations to deliver EBM lectures, moderate journal clubs, and conduct training in patient communication. We also saw an increased demand for HSO residents by a number of Russian medical institutions. All HSO residents (n = 27) have graduated from official Russian residency, successfully employed as oncologists according to their subspecialty with 71% (n = 19) in high-volume oncology hospitals. Learning how to expand the project under conditions of resistance was an essential element in implementing new educational strategies.

This study has several limitations related to retrospective design. First, we were unable to provide the results of the patient communication exam for all HSO residents, as it was conducted for only two resident enrollments during PGY2, because of cost of simulation with actors and independent expert assessment. Second, there is potential selection bias between the groups of communication assessment as HSO residents had passed a rigorous three-step selection before enrollment; therefore, communication baseline skills could be higher. Third, there was no long-term assessment of the programs’ impact because of only the 5-year follow-up. Fourth, the cross-sectional evaluation of EBM knowledge can be biased by different baseline levels at enrollment and longitudinal program assessment with pre- and postprogram evaluation is needed. Prospective studies with longer surveillance are needed to overcome these limitations.

In conclusion, Western-trained expatriates involved in remote oncology training improve EBM knowledge and enhance PCS among young oncologists in their home country. This strategy allows for the creation of a sustainable program and can be efficient in circumstances where EBM and PCAs are not widespread and there is a lack of mentors practicing them. We believe that programs like the HSO can provide systemic changes in cancer care in developing countries and can be replicated in other specialties.
